# Research on a Critical Link Discovery Method for Network Security Situational Awareness

**DOI:** 10.3390/e26040315

**Published:** 2024-04-04

**Authors:** Guozheng Yang, Yongheng Zhang, Yuliang Lu, Yi Xie, Jiayi Yu

**Affiliations:** 1 College of Electronic Engineering, National University of Defense Technology, Hefei 230037, China; yangguozheng17@nudt.edu.cn (G.Y.); luyuliang@nudt.edu.cn (Y.L.); xie_yi@nudt.edu.cn (Y.X.); yujiayi_yjy@nudt.edu.cn (J.Y.); 2 Anhui Province Key Laboratory of Cyberspace Security Situation Awareness and Evaluation, Hefei 230037, China

**Keywords:** network security situational awareness, critical link, multi-layer network, mapping entropy

## Abstract

Network security situational awareness (NSSA) aims to capture, understand, and display security elements in large-scale network environments in order to predict security trends in the relevant network environment. With the internet’s increasingly large scale, increasingly complex structure, and gradual diversification of components, the traditional single-layer network topology model can no longer meet the needs of network security analysis. Therefore, we conduct research based on a multi-layer network model for network security situational awareness, which is characterized by the three-layer network structure of a physical device network, a business application network, and a user role network. Its network characteristics require new assessment methods, so we propose a multi-layer network link importance assessment metric: the multi-layer-dependent link entropy (MDLE). On the one hand, the MDLE comprehensively evaluates the connectivity importance of links by fitting the link-local betweenness centrality and mapping entropy. On the other hand, it relies on the link-dependent mechanism to better aggregate the link importance contributions in each network layer. The experimental results show that the MDLE has better ordering monotonicity during critical link discovery and a higher destruction efficacy in destruction simulations compared to classical link importance metrics, thus better adapting to the critical link discovery requirements of a multi-layer network topology.

## 1. Introduction

As the structure of the internet becomes increasingly complex and large, security threats in cyberspace present multi-dimensional, large-scale, and highly destructive behavioral characteristics and penetrate into all levels of physical devices, business applications, and user roles. In the field of network security situational awareness (NSSA), technical personnel perform network situational awareness and threat analyses and implement targeted security policies based on the network topology to ensure the secure operation and maintenance of the entire network environment. They may use various technical means, such as traffic monitoring, intrusion detection, honeypots, vulnerability scanning, and other related tools, to accomplish their tasks [1,2,3,4].

At the same time, a significant number of scholars have centered their research around assessment methods in network security situational awareness. Chen et al. [5] used AHP correlation analysis to construct a quantitative assessment model of cyber threats involving various aspects such as the system, host, and service. Further, Kong et al. [6] proposed a comprehensive evaluation model combining the hierarchical analysis idea and the fuzzy evaluation method. In addition, to increase their effectiveness, theories such as rough set theory and cluster analysis [7,8] have been introduced into the design of situational assessment methods.

All of the above situation assessment models use network topology information as the backbone of analysis. However, currently, network topology information acquisition and analysis mainly focus on the interconnection level of network devices, and in our previous work [9], the limitations of this kind of assessment relying on a single-layer network basemap are elaborated, which mainly consist of the following four aspects: (a) Single-layer network topology models cannot characterize the formation of business correlations between network applications based on data traffic. (b) Single-layer network topology models are unable to characterize the interpersonal associations between network users based on business access and communication interactions. (c) Threat correlation analysis by purely device-layer networks is inadequate after the discovery of anomalous network behavior. (d) Single-layer network topology models provide insufficient support for critical node identification and critical link discovery, and the networks’ structural information is monolithic. To overcome the limitations of single-layer networks in situational analysis, we propose a multi-layer network model for network security situational awareness.

As an analytical framework that can comprehensively characterize the hierarchical coupling relationship of complex systems, multi-layer networks [10,11] have been gradually introduced into situational awareness models in various fields. In cyberspace, according to the different application scenarios, the component properties and connection modes of various types of networks have different characteristics. For example, the physical device layer (labeled PD), which is embodied in the routing relationship, corresponds to communication connections between physical devices such as servers, terminals, and routers. The business application layer (labeled BA), embodied in the access relationship, corresponds to the business intertwining between different business systems and access nodes. The layer of user roles (labeled UR) embodied in online social networking corresponds to diverse associative relationships between virtual account roles, and multi-layer networks are capable of inscribing patterns of connectivity between multiple networks simultaneously.

In previous work [12], we focused on identifying critical nodes in multi-layer network models. To achieve this, we propose a method that combines multi-attribute decision making and node-dependent mechanisms. The experiments show that this fusion method performs better in terms of evaluation performance compared to the classical node identification method [13,14,15,16].

In network security situational awareness, the critical link discovery method is another research focus that corresponds to the critical node identification method. For the multi-layer network model, link importance is measured in terms of both topology and dependencies. Links in a network are characterized as various types of connectivity relationships between nodes at the time of model construction. Compared with nodes, links have outstanding characteristics: On the one hand, links in the network are more extensive, and they have more diverse types. At the same time, a single link has less impact on the network, so the effect is not obvious enough in simulation experiments. On the other hand, they are limited by data sources and detection methods, and compared with the nodes, the integrity of the information of the links may be worse, so local information assessments are more urgent. Given this, by fusing the entropy of link-local betweenness mapping with the link-dependent mechanism in multi-layer networks, this paper proposes a link importance assessment metric that builds an analytical domain around the target link: the multi-layer-dependent link entropy (labeled MDLE). The main contributions of this work are as follows:

- Our work introduces mapping entropy into the link importance assessment method for the first time, which effectively expands the domain of link importance assessments by fusing the topological information of links with neighborhood set information, thus increasing the assessment efficacy of relevant link discovery metrics.
- Based on the dependent mechanism of the multi-layer network model, this paper proposes a critical link discovery metric, multi-layer link mapping entropy (MDLE), which is more adaptable to the link assessment needs of multi-layer networks than the classical link discovery metrics and has more advantages in assessment accuracy and network damage effects.

## 2. Related Work

In this section, the research work related to critical link discovery is summarized.

### 2.1. Critical Link Discovery

The research on critical link discovery methodologies is an incremental process, with the migration of node importance metrics as part of the early link importance assessment metrics. By improving the node betweenness centrality proposed by Freeman [13], Newman [17] proposed link betweenness centrality. The link degree [18] as a migration method for node degree has been applied to several research areas such as link importance assessments as well as link prediction. Sun et al. [19] constructed a k-core index variant for links by introducing the k-core decomposition theory into link importance assessments, which made some improvements to the assessment efficiency. However, their study only focuses on single-layer complex networks and does not incorporate the unique dependency characteristics of multi-layer networks to adapt to a wider and finer range of assessment needs. Chen et al. [20] measured the importance of nodes and edges in network analyses by designing functions that calculate the additive edge priority, node degree, and median centrality, but this focuses on how to efficiently identify and quantify the importance of individual components in a network. Onnela et al. [21] proposed the topological overlap index to analyze the performance characteristics of communication networks in the face of link removal in the study of the local and global structure of societal communication networks. However, it is limited to a single neighborhood analysis, focusing on the neighborhood and overlaps between nodes in a single-layer network. Kimura [22] solved the problem of minimizing the spread of computer viruses or malicious rumors based on a natural greedy strategy to efficiently find a finite number of links in a network. To address the problem of large link sizes and expensive computations, Saito et al. [23] tackled this problem by using the bottom-k sketch algorithm and further by employing two new acceleration techniques: marginal link updating (MLU) and redundant link skipping (RLS). In subsequent work, Saito [24] introduced marginal node pruning (MNP) and burn out following (BOF) to further accelerate the efficiency of network link evaluations. To address the problem of how to effectively identify critical links that severely degrade the network performance, Kazumi et al. [25] utilized bridge detection techniques in graph theory to efficiently identify critical links in the context of node reachability as a performance measure. However, none of the survey papers mentioned above consider the problems of multi-layer networks, let alone the connectivity characteristics of links at different levels, dependencies across layers, efficient transfer, and merging of link importance, which is exactly what we propose.

### 2.2. Type of Link

It is worth noting that the exploration of the nature of links in multi-layer networks has also been receiving a lot of attention. Links with direction can characterize the directionality of information or paths, while links with weights can effectively characterize the disparity in the amount of information contained between links [26]. Based on the topological distribution, it was found that some links exist only between nodes belonging to different sets (e.g., bipartite networks) [27], acting as intermediary connecting nodes of different natures. At the same time, some links have a temporal dimension, are active in performing their functions only in a specific time interval, and are in a disconnected state at non-specific times [28]. Mikko [29], in their survey on multi-layer networks, describes the location of links according to their topological position, categorizing them as “intra-layer” or “inter-layer”. Buldyrev [30] explored multiple interdependent networks in many systems in society, from engineering to healthcare, where nodes are functionally interdependent and therefore links are also described as media capable of transmitting functional dependencies. This design inspired the idea of transmitting the importance of the components of the three-layer networks in this paper.

In summary, the discussion on importance assessments of links and link types cuts across several fields, which provides a solid foundation for related research work in the field of network security.

## 3. Materials and Methods

### 3.1. Network Model Definition

In this section, the relevant network model definitions are described. To begin, let us provide the formal definition of a single-layer network.

**Definition** **1** (Single-Layer Network). *A graph (i.e., a single-layer network) is a tuple G=(V,E), where V is the set of nodes and E⊆V×V is the set of edges that connect pairs of nodes.*

Similarly, we introduce the formal definition of a multi-layer network.

**Definition** **2** (Multi-Layer Network). *A multi-layer network M is represented by a binary M=(G,C), where the network $G=\left\{G^{\alpha };\alpha \in \left\{1,\cdots ,l\right\}\right\}$ is the collection of (directed or undirected, entitled or un-empowered) graphs $G^{\alpha }=\left(V^{\alpha },E^{\alpha }\right)$ and $G^{\alpha }$ is called the αth layer of the multi-layer network M. $C=\left\{E^{\alpha \beta }\in V^{\alpha }\times V^{\beta };\alpha ,\beta \in \left\{1,\cdots ,l\right\},\alpha \neq \beta \right\}$ is a collection of inter connections between nodes of different layers $G^{\alpha }$ and $G^{\beta }\left(\alpha \neq \beta \right)$. Element $E^{\alpha }$ is called intra-layer connections of the multi-layer network M, while element $E^{\alpha \beta }\left(\alpha \neq \beta \right)$ is called inter-layer connections.*

### 3.2. The Multi-Layer Network Model for Network Security Situational Awareness

Current network security situation assessment models rely on the single-layer network structure to sort out and analyze the inventory of assets and vulnerability information in the network to set up a risk-level assignment strategy for the entire network environment. However, this network structure ignores the vertical influence of business systems and user groups in security assessments, resulting in the whole assessment behavior being confined in a flat framework. To address this problem, our team proposed a multi-layer network model for network security situational awareness based on the literature [9]. The model is broadly described as follows.

**Figure 1:**
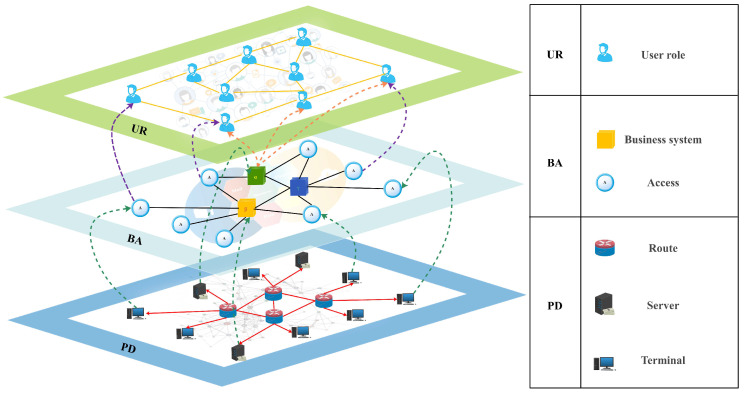
Schematic diagram of multi-layer network architecture. (**bottom**) The physical device layer (labeled as PD) demonstrates the routing communication relationship, which contains the communication connections between physical devices such as servers, terminals, and routers. (**middle**) The business application layer (labeled as BA) shows the business access relationship, which contains the business intertwining between different business systems and access nodes. (**top**) The user role layer (labeled as UR) corresponds to various association relationships between virtual account roles in online social networks.

According to the construction logic of the current internet structure, the physical level of network equipment deploys various types of business system nodes and access nodes. In contrast, user roles within the business system form a social group at the internet level due to business associations. Based on the mapping relationships of the internet at different levels, we construct a multi-layer network model by aggregating the three main networks that maintain the normal flow of information on the internet: the physical device layer network (PD), the business application layer network (BA), and the user role layer network (UR), as shown in Figure 1. This network model will be described below from three perspectives: network architecture layering, the node bearer medium, and link connection types.

#### 3.2.1. Network Architecture Layering Perspective

The information for the different network layers in this model is derived from the different mapping domains in which the internet service operates. The model is divided into three network layers from the bottom up: the physical device layer, the business application layer, and the user role layer. The physical device layer is the physical support for the operation of the entire network system, providing the operating environment for business access. The business application layer portrays the access relationship of the application business flow, and the users belonging to each business system constitute the network social group. The user role layer describes the user group’s social relationship and business association in the network.

This means that the physical device layer network assigns the communication entity connections as the underlying support for information transmission and data flow. The business application layer network describes the intertwining and correlation of services and contains business access systems to meet the application needs of various types of users. The user role layer portrays the social structure in cyberspace and characterizes online social groups with account association properties.

#### 3.2.2. Node Bearer Medium Perspective

As a multi-dimensional portrayal model of network situations, the nodes in the multi-layer network can be classified into six categories of heterogeneous nodes based on their topological location and functional attributes.

There are three types of heterogeneous nodes in the physical device layer network: router nodes, server nodes, and terminal nodes. The router node in the inner layer of the network is responsible for communication hopping and coordination. The server node at the edge of the network provides the operation and maintenance environment for the business systems, and the terminal node is the physical interface for ordinary users to log into the network.

There are two types of heterogeneous nodes in the business application layer: business system nodes and access nodes. The business system node mainly carries all kinds of business flows in the network, approves access, and provides corresponding services, and the access node is the mapping identifier of the terminal entity devices in the business access relationships.

There exists a type of heterogeneous node in the user role layer, i.e., the user node, which mainly characterizes all kinds of accounts and user IDs generated by relying on the business system. It is the access credentials for users to apply for application services from all kinds of business systems.

#### 3.2.3. Link Connection Type Perspective

Links in the multi-layer network can be categorized as an “intra-layer link” or an “inter-layer link” in terms of their topological location. However, in the process of network research, we tend to pay more attention to the role of the link in terms of the network-specific aim, that is, the link connection type. So, for classification with regard to the network link connection type, the network contains two types of links: “connected links” and “dependent links”.

A connected link describes an undirected connected relationship between nodes, indicating the presence of connectivity and association characteristics between nodes, but they do not have a dependent relationship. Dependent links, based on characterizing the connectivity between nodes, imply that nodes are not pairwise equivalent, but rather have primary and secondary dependencies, and directed links are used to characterize this dependent relationship. The endpoints of dependent links are divided into “support nodes” (located at the start of the directed link) and “dependent nodes” (located at the target end of the directed link). The support node provides the deployment environment for the dependent point connected to it or plays a direct control role; when all the support nodes fail, it will cause the dependent node of the following one to fail. A dependent node has no direct effect on the state of the relevant support nodes or the type of connection between the nodes, as the description in Table 1 shows.

## 4. Critical Link Discovery

### 4.1. Problem Analysis

Analyzing the connection pattern of the above multi-layer network model, it can be found that the links in the model contain two categories: “connected links” and “dependent links”. A connected link has a general connectivity role between nodes, characterizing the existence of connectivity between nodes and using an undirected link as an identifier. The nodes at the two ends of the dependent link are defined as the dependent node and the support node, respectively. While playing the role in the connectivity of nodes, dependent links also implement the following mechanisms: when a support node fails, the dependent node associated with it will also experience a secondary failure; similarly, the dependent node will fail when the dependent link is disconnected. The dependent link in the model is identified using a directed link.

It is important to note that while there has been extensive research on critical node identification methods in complex networks in recent years, the research on critical link discovery has been severely limited thus far. Unfortunately, most of the approaches taken are based on the direct or indirect expansion of node identification methods, which do not properly consider the importance of the link as the central object of research. Rather, they solely focus on evaluating the importance of the two endpoints of the link and fusing them separately, which is a major limitation that must be addressed. In conjunction with the multi-layer-dependent network model mentioned above, the following main requirements need to be met when designing a critical link discovery method. (The four requirements are our abstract assumptions, and are solely the ones we care most about from cybersecurity and practical need perspectives, so they are not comprehensive. There is no doubt that other extensions are possible, so we will continue to iterate and update them continuously in subsequent work).

(a) The size of the links in the network will increase at a significant rate compared to the nodes, and even in sparse graphs, the number of links may be much larger than the nodes. It is much more difficult for the network to collect all the information on links than to collect information on nodes. Therefore, when conducting a critical link importance assessment, it should be centered around the local information of the links as much as possible.
(b) During the discovery of a critical link, if many links have the same importance score, then it will not be possible to make a precise importance decision, so the importance assessment metrics for links need to have a high granularity for assessment.
(c) Since the model has two types of links, “connected links” and “dependent links”, the contribution of the target link in both connected and dependent relationships should be considered to ensure the generalizability of the link identification methodology (both types can be evaluated using the same framework).
(d) The critical links obtained according to the critical link identification method should have a certain status in the network structure and undertake important functional tasks, and will have a large impact on the network when the critical links are removed or fail.

To meet the above assessment needs, this paper proposes a link importance assessment metric called MDLE, which integrates the link betweenness centrality in the first-order central domain and the mapping entropy to comprehensively assess the importance of links. Next, the following classic metrics will be presented for assessing the importance of a link.

### 4.2. Classical Critical Link Assessment Metrics

This section presents the definitions and descriptions of classical critical link assessment metrics. The betweenness centrality [17] of link *e* is denoted as ${\mathrm{LBC}}_{e}$ and defined as follows.

**Definition** **3** (Link Betweenness Centrality).

(1) $${\mathrm{LBC}}_{e}=\sum_{s,t\in v}\frac{\sigma (s,t|e)}{\sigma (s,t)}$$

*In Equation (1), s and t are any two nodes in the network that are not identical, σ(s,t) characterizes the number of shortest paths that exist between node s and node t, and σ(s,t∣e) characterizes the number of cases in the shortest path that pass through link e, where σ(s,t∣e)=1/n when the shortest path length is n first-order links.*

The link betweenness centrality is considered to be a better choice for evaluating the importance of links with a high accuracy and strong discrimination, but this method is complicated to compute and not easy to apply due to the difficulty in grasping global information.

The Jaccard index [31] of link *e* is denoted as ${\mathrm{Jaccard}}_{e}$ and defined as follows.

**Definition** **4** (Jaccard Index).

(2) $${\mathrm{Jaccard}}_{e}=\frac{\left|U_{x}\cap U_{y}\right|}{\left|U_{x}\cup U_{y}\right|}$$

*In Equation (2), x and y characterize the two end nodes of link e, $U_{x}$ characterizes the set of neighboring nodes of node x, and $U_{y}$ characterizes the set of neighboring nodes of node y. A smaller value of the Jaccard index indicates that the degree of dissimilarity between the two endpoints of link e is higher and the importance of the link is stronger.*

The degree centrality [18] of link *e* is denoted as ${\mathrm{LDC}}_{e(i,p)}$ and defined as follows.

**Definition** **5** (Link Degree Centrality).

(3) $${\mathrm{LDC}}_{e(i,p)}=\frac{\sum_{j}^{N}x_{ij}\cdot \sum_{q}^{N}y_{pq}}{{\left(N-1\right)}^{2}}$$

*In Equation (3), N is the total number of nodes in the network, i and p characterize the two end nodes of link e, respectively, j characterizes the other nodes in the network except node i, and $x_{ij}$ is the connection state vector of node i to node j, which is 1 when there is a connection and 0 otherwise. Similarly, q characterizes the other nodes in the network except node p, and $y_{pq}$ is the connection state vector of node p to node q, which is 1 when there is a connection and 0 otherwise.*

Link degree centrality can fit the connection weights of link endpoints with a low computational complexity, but only local information about the size of neighboring nodes is taken into account, the computational granularity is coarse, and the importance is not well identified.

The k-shell index [19] of link *e* is denoted as ${\mathrm{LKS}}_{e}$ and defined as follows.

**Definition** **6** (Link K-shell Index).

(4) $${\mathrm{LKS}}_{e_{\left(i,j\right)}}^{\max}=\max\left\{{\mathrm{KS}}_{i},{\mathrm{KS}}_{j}\right\}$$

(5) $${\mathrm{LKS}}_{e_{\left(i,j\right)}}^{\min}=\min\left\{{\mathrm{KS}}_{i},{\mathrm{KS}}_{j}\right\}$$

(6) $${\mathrm{LKS}}_{e_{\left(i,j\right)}}^{\mathrm{pro}}={\mathrm{KS}}_{i}*{\mathrm{KS}}_{j}$$

*In Equations (4)–(6), ${\mathrm{KS}}_{i}$ and ${\mathrm{KS}}_{j}$ characterize the k-shell indices of the two endpoints of link $e_{\left(i,j\right)}$, and ${\mathrm{LKS}}_{e_{\left(i,j\right)}}^{\max}$, ${\mathrm{LKS}}_{e_{\left(i,j\right)}}^{\min}$, and ${\mathrm{LKS}}_{e_{\left(i,j\right)}}^{\mathrm{pro}}$ are the link shells under different fitting strategies, respectively. The link shells characterize the link’s importance by the degree of centrality in the network of endpoints connected by the current link.*

The topological overlap index [21] of link *e* is denoted as $\mathrm{TO}\left(e_{\left(i,j\right)}\right)$ and defined as follows.

**Definition** **7** (Topological Overlap Index).

(7) $$\mathrm{TO}\left(e_{\left(i,j\right)}\right)=\frac{n_{ij}}{k_{i}-1+k_{j}-1-n_{ij}}$$

*In Equation (7), $n_{ij}$ describes the number of common neighbors of node i and node j. The existence of common neighbors of node i and node j makes the following possible when the link is blocked: information can still be propagated through the paths formed by common neighbor nodes and the higher the number of replaceable links, the lower the importance of the link.*

### 4.3. Critical Link Discovery Method Fusing the Link-Local Betweenness Mapping Entropy and the Link-Dependent Mechanism

#### 4.3.1. Link-Local Betweenness Centrality

In existing research, the discovery methods for critical links are mainly divided into two aspects: One is the numerical fitting of the critical node identification results, for instance, the link degree centrality (LDC) and link k-shell index (LKS). The other is the formal transformation of the critical node discovery methods, for instance, the link betweenness centrality (LBC), the Jaccard index, and so on. However, it can be seen that none of the above methods have established an important analysis framework specifically for the target links.

Previously, for the neighborhood importance information extraction from links, the method used was numerical fitting based on analyzing the importance information of the two end points of the target link. However, for the target link, there is a natural overlap between the neighborhoods of the two end points, which affects the judgment of the important information of the link. Therefore, we believe that the analysis domain for links should be established with the target link as the central object.

In graph theory, analogous to the notion of node neighborhood, the link neighborhood is defined as follows.

**Definition** **8** (Link Neighbor). *In the graph G=(V,E), let e be a link in the graph, i.e., e∈E. The open neighborhood of a link e in G refers to the open neighborhood set ${\mathrm{EN}}_{G}\left[e\right]=\{f\in E|f\neq e,f$ is the neighbor of e}, and the closed neighborhood of a link e in G refers to the set $M_{G}\left[e\right]=\left\{e\right\}\cup {\mathrm{EN}}_{G}\left[e\right]$.*

**Figure 2:**
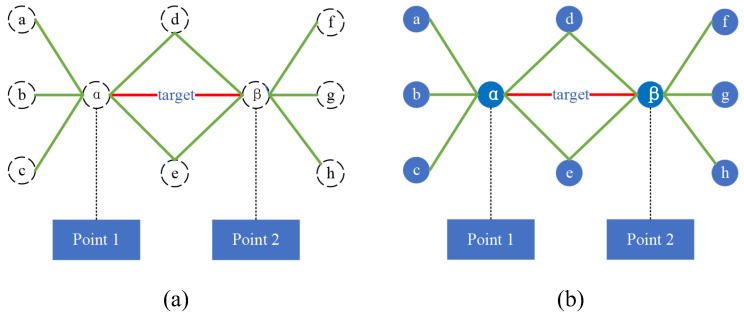
Schematic diagram of link neighborhoods and first-order central domains. (**a**) It represents the link neighborhoods, which is defined as the set of links, i.e., it does not contain nodes, and therefore the nodes in (**a**) are represented by circles surrounded by dashed lines. (**b**) It represents the link first-order central domain, the link first-order central domains is defined as a two-element set (nodes and links) containing both nodes and links, hence the nodes in (**b**) are represented by solid circles.

Link *e* is considered to be destroyed in G when both endpoints of link *e* and its closed neighborhood $M_{G}\left[e\right]$ are removed from the network; accordingly, for a subset of links S⊆E(G), S is said to be a link destruction strategy in G if every link in S is destroyed. When every link in S is destroyed, the resulting subgraph of G is called a surviving subgraph, denoted G/S, and the link neighborhood is shown in Figure 2a.

Since the concept of a link neighborhood does not include all the nodes of neighboring links, i.e., the blue nodes (a,b,…,h), which is not conducive to the computation of local centrality in the network, this paper derives the concept of a link neighborhood and proposes the “link first-order central domain” as follows, whose structure is shown in Figure 2b.

A link neighborhood is defined as a collection of links, and the nodes in Figure 2a are represented because the nodes are not included in the link neighborhood, but only as associated nodes between links. A link first-order central domain is defined as a two-element set (node and link) that contains nodes and links, and the nodes in Figure 2b are represented by solid circles because the nodes are contained in the link first-order central domain.

**Definition** **9** (Link First-order Central Domain). *In graph G(V,E), let $M_{G}\left[e\right]$ be the closed neighborhood of link e. The first-order central domain of link e can be defined as ${\mathrm{FC}}_{G}\left[e\right]=\left\{M_{G}\left[e\right]\cup I|I\right.$ and is the set of endpoints of e and the neighboring links of $\left.e\right\}$. It can be seen that the link first-order central domain is a local analysis domain built around link e.*

Link betweenness centrality is considered an essential metric with good evaluation results. Still, the applicable scenarios are limited because they require complete topological network information to build the analysis domain for calculation. In the link first-order centroid domain, analogous to the notion of the self-network mediator of a node in a node’s neighborhood, the link-local betweenness centrality (labeled LLBC) in the link first-order central domain can be proposed as an importance metric for links.

**Definition** **10** (Link-Local Betweenness Centrality).

(8) $${\mathrm{LLBC}}_{e}=\sum_{s,t\in {\mathrm{FC}}_{G}\left[e\right]}\frac{\sigma (s,t|e)}{\sigma (s,t)}$$

*where nodes s and t belong to the first-order central domain of link e. The local betweenness centrality of a link can effectively assess the importance of the target link in the first-order central domain in terms of communication functionality, and the method only requires local information about the network.*

#### 4.3.2. Link-Local Mapping Betweenness Entropy

It was found through an analysis of the above ideas about LLBC that although the LLBC solves the problem of the strong dependence of global metrics on the completeness of topological information, the scope of the analysis of link topological centrality is also limited to the one-hop range of the target link. To further extend the scope of the analysis domain, the concept of mapping entropy is introduced in this paper.

The idea of entropy was first introduced by Clausius. Initially, it was used as a parameter for determining the state of matter and describing the process of “energy degradation”. However, as entropy became more widely applied across different fields, researchers began to attribute more specific characteristics to it. One such characteristic is information entropy, proposed by Shannon in the area of information theory. Information entropy is commonly used in complex network analyses, where it serves as a measure of information variation. Higher entropy values indicate greater variation in the metric information, which in turn carries less information. Conversely, lower entropy values signal more stable metric evaluations, with more information content. The definition of network information entropy is as follows.

Given a graph G(*V*, *E*), define the node information entropy $E_{i}$ as follows.

**Definition** **11** (Information Entropy).

(9) $$E_{i}=-\sum_{i}I_{i}\log I_{i}=-\sum_{i}{\mathrm{DC}}_{i}\log{\mathrm{DC}}_{i}$$

*In Equation (9), $I_{i}$ is the importance degree of node/link i. It can be replaced with metrics such as degree centrality, betweenness centrality, etc., and node degree centrality (labeled DC) is used here as an example.*

Nie et al. [32] proposed that the information entropy fusion can be used to assess the amount of information of the target (node/link) within the neighborhood range, and further extended the analysis domain to obtain the concept of mapping entropy. The related definitions are as follows.

The mapping entropy of node *i* is denoted as ${\mathrm{ME}}_{i}$ and defined as follows.

**Definition** **12** (Mapping Entropy).

(10) $${\mathrm{ME}}_{i}=-{\mathrm{DC}}_{i}\sum_{i}\log{\mathrm{DC}}_{j}$$

*In Equation (10), ${\mathrm{DC}}_{i}$ is the degree centrality of the target node i and j belongs to the neighborhood set of node i.*

The mapping entropy can comprehensively measure the amount of information of the target component as well as the neighbor component of the assessment metrics within the scope of the analysis domain and has achieved better application results in research.

Analyzing the construction of the mapping entropy, it can be found that this metric simultaneously integrates the importance score of a certain indicator itself and the score of the neighboring objects under the same evaluation index, so that through the mechanism of mapping entropy, we can extend the analysis scope of the link-local betweenness centrality to the next-hop neighborhood, and further synthesize more topology information for the analysis of the importance of the target link.

We establish the link-local mapping betweenness entropy (labeled LLBME) in the first-order central domain by examining the connection between a link and its neighboring links, much like the node mapping entropy.

This is determined by combining the link-local betweenness centrality of the link and its neighboring links, as defined below.

**Definition** **13** (Link-local Mapping Betweenness Entropy).

(11) $${\mathrm{LLBME}}_{e_{1}}=-{\mathrm{LLBC}}_{e_{1}}\sum_{e_{1}}\log{\mathrm{LLBC}}_{e_{2}}$$

*In Equation (11), ${\mathrm{LLBC}}_{e_{1}}$ is the local betweenness centrality of link $e_{1}$ in the first-order centroid domain, and ${\mathrm{LLBC}}_{e_{2}}$ is the local betweenness centrality of link $e_{1}$’s neighboring link $e_{2}$.*

#### 4.3.3. Link-Dependent Mechanism

**Figure 3:**
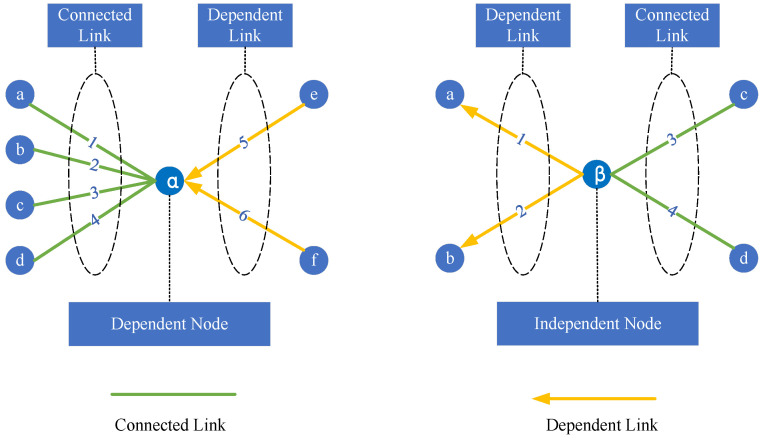
Schematic diagram of the node-dependent mechanism.

As shown in Figure 3, when studying the dependency relationships between nodes, the analysis domain is constructed with the nodes as the core, and the nodes are categorized as a “dependent node” or an “independent node” according to whether they need the support of other nodes or not. The set of “dependent links” characterizes the dependencies between nodes, while the set of “connected links” represents the connectivity between nodes. Dependent nodes must rely on other nodes for their survival, while “independent nodes” are characterized as functioning without the support of other nodes.

**Figure 4:**
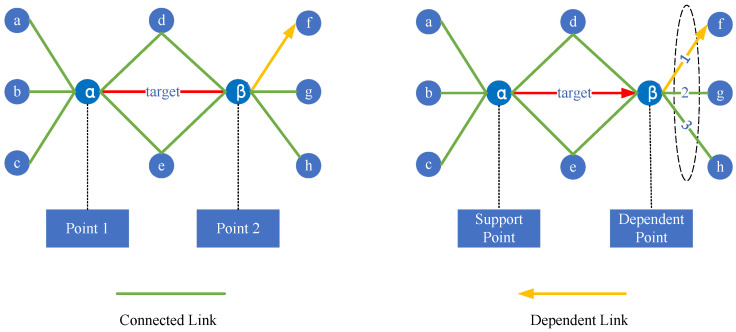
Schematic diagram of the link-dependent mechanism.

When the analysis domain is established with the target link as the center, as shown in Figure 4, and the analyses of “connected links” and “dependent links” are conducted separately, it can be concluded that:

(a) The analysis of “connected links”, whose two endpoints α and β are on an equal footing (Points), which carry out the connectivity function in the network, shows that they are substitutable and do not form a dependency with their neighboring links.
(b) Analyzing the “dependent links”, its two endpoints α and β have different roles (support node and dependent node), and there is a dependent relationship between the source endpoint α and the target endpoint β. In other words, when the source endpoint α fails or the link $e_{\left(\alpha ,\beta \right)}$ is broken, the target endpoint β will also fail, and the other links also dependent on the target endpoint β will also be broken. Therefore, from the perspective of node importance, the importance of the target endpoint β needs to be transmitted to the source endpoint α. Similarly, from the perspective of link importance, the importance of the target endpoint β’s neighboring links other than the homologous dependent links also needs to be transmitted to link $e_{\left(\alpha ,\beta \right)}$.

In summary, fusing the link-local betweenness mapping entropy in the first-order central domain with the link-dependent mechanism yields the multi-layer-dependent link entropy (MDLE).

**Definition** **14** (Multi-Layer-Dependent Link Entropy).

(12) $${\mathrm{MDLE}}_{e_{1}}={\left({\mathrm{LLBME}}_{e_{1}}\right)}^{-1}+\sum_{e_{2}}\;S_{e_{2}}$$

*In Equation (12), ${\mathrm{LLBME}}_{e_{1}}$ characterizes the link-local betweenness mapping entropy in the first-order central domain, and since the values of the entropy-like metrics are negatively correlated with the importance of the link, the inverse is taken here as the primitive score of the link’s importance. $S_{e_{2}}$ then denotes the score of the importance of the rest of the neighboring links, other than the same-direction dependency links, that are connected to the target endpoint β of link $e_{1}$ when link $e_{1}$ is a dependent link.*

#### 4.3.4. Critical Link Discovery Process

To summarize, the critical link discovery metric, the MDLE, will be able to transfer the link importance score based on the decomposition of the network coupling relationship according to the dependency relationship of links and determine the link connectivity importance and dependent importance for the discovery of critical links in the multi-layer dependent network.

The methodological process for the MDLE consists of five phases:

- S1: network input phase.

Based on the target network data, the set of nodes and links is extracted and the network topology is constructed.

- S2: link-local betweenness computation phase.

Construct the first-order central domain of the target link, compute the local link median, and evaluate the importance of the target link.

- S3: mapping entropy calculation phase.

The corresponding mapping entropy is computed based on the link-local betweenness centrality of the target link, extending the scope of the analysis domain.

- S4: comprehensive link importance assessment phase.

Determine the fusion mapping entropy based on link dependencies to compute link importance in the multi-layer network.

- S5: sequence output phase.

**Figure 5:**
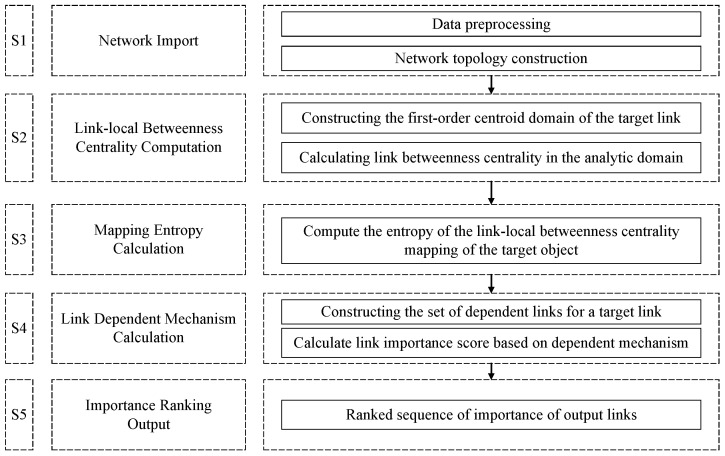
MDLE critical link discovery process.

Output the sequence of critical links based on link importance. The flow chart is detailed in Figure 5.

## 5. Network Experiment and Result Analysis

### 5.1. Dataset and Experimental Setup

To evaluate the critical link discovery method proposed in this paper, we tested it on two multi-layer networks: a “typical business network” (labeled Business) and a “typical campus network” (labeled Campus) [33]. Both networks have three-layer architectures: a physical device layer, a business application layer, and a user role layer. The datasets were created using the heuristic algorithm from the work [9], which establishes a “one-to-one” dependency between the physical device layer and the business application layer based on the area routing topology data. This means that if a server node fails, its corresponding business system node also fails. If an access node fails, its corresponding user terminal also fails. There are also multi-layer dependencies between the business application layer and the user role layer, and “many-to-many” dependencies between them. This means that after the failure of a business system node or an access node, the corresponding user nodes will immediately fail.

To further validate the effectiveness of the approach, in addition to the critical link discovery metric MDLE, five existing link importance metrics, i.e., the link degree centrality (LDC), link betweenness centrality (LBC), link k-shell index (LKS), Jaccard index (Jaccard), and topology overlap index (TO), will be used as an experimental control group in the experiments in this section.

Similar to the idea of node importance assessments, in the multi-layer network topology model for network security situational awareness, starting from the idea of the MDLE, the importance of the links in the upper two layers of the three-layer network, as well as the importance of inter-layer links, will be summarized in the physical device layer, which will become the basis for judging the importance of various types of links in the physical device layer. These important links in the physical device layer will play a critical role in supporting the structure of the entire network topology. Therefore, when using various types of non-neighborhood-centric metrics (LBC, LKS) to generate link importance ranking sequences, we still set the evaluation scope at the physical device layer to ensure the consistency of the evaluation level.

Next, the experimental part will comprehensively analyze the MDLE from three perspectives: monotonicity rankings, metric correlations, and network destruction effects.

### 5.2. Link Importance Metric Ranking Monotonicity

Ranked monotonicity [34] is an important metric for performance evaluations of critical link discovery methods; a higher ranked monotonicity implies a smaller size of the set of medium volume scores for link output sequences, a weaker ambiguity in importance decision making, and a finer granularity of importance evaluations.

**Definition** **15** (Link Monotonicity).

(13) $$M_{r}^{p}\left(e\right)=\left(1-\frac{\sum_{r\in R}\;L_{r}\left(L_{R}-1\right)}{L^{p}\left(L^{p}-1\right)}\right)$$

*In Equation (13), the set of to-be-evaluated monotonicity links is selected using sampling, and p represents the proportion of the subset of to-be-evaluated links selected from the network links to the total number of links, R is the set that divides the to-be-evaluated links into sets conditioned on the condition that they have the same scores, $L^{p}$ is the size of the set of to-be-evaluated links, and $L_{r}$ is the number of links in the to-be-evaluated link sets that have the same scores. The score of the respective groups of links with the same scores is r.*

**Figure 6:**
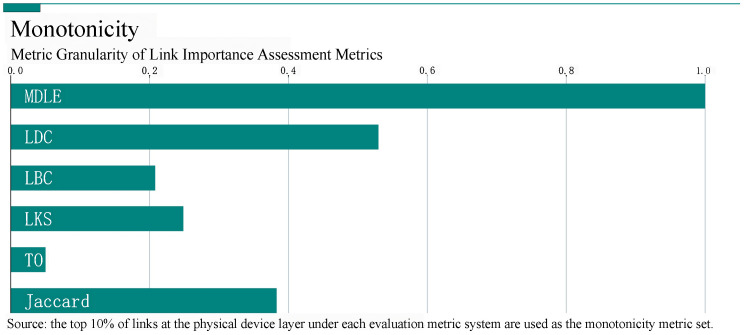
Schematic of monotonicity ranking of link importance metrics.

In this section, the experiment is based on the network topology of the dataset “Business” network. We choose the top ten percent of the links in the physical layer of the network layer in the rankings of the MDLE and the other five metrics (LDC, LBC, LKS, Jaccard, and TO) as the set of links to be evaluated. The monotonicity of the metrics is shown in Figure 6.

The experimental results show that compared with the other five metrics, the monotonicity of MDLE can be maintained at 1.0, while the monotonicity of the rest of the metric rankings is less than desirable, which is because the structural homogeneity of links is more obvious than that of nodes considering the scale and the network characteristics. The MDLE not only takes into account the degree of influence of the links in the homogeneous range, but also combines the dependent mechanism with the synthesis of the links in the multi-layer networks. Importance information makes the source of evaluation information more diversified and solves the problem of the low decision-making accuracy in existing link importance metrics.

### 5.3. Link Importance Metric Correlation Analysis

To further analyze the correlation between the MDLE and the existing link importance metrics in terms of ranking, the Pearson product-moment correlation coefficient (labeled PPMCC) [35] is used here to calculate the correlation between the metrics used in this section (MDLE, LDC, LBC, LKS, Jaccard, TO). The Pearson correlation coefficient is defined as follows.

**Definition** **16** (Pearson Product-Moment Correlation Coefficient).

(14) $$\gamma =\frac{1}{n-1}\sum_{i=1}^{n}\left(\frac{X_{i}-\bar{X}}{\sigma_{X}}\right)\left(\frac{Y_{i}-\bar{Y}}{\sigma_{Y}}\right)$$

*In Equation (14), for the set of samples X and Y of the two link importance metrics for correlation assessment, $\frac{X_{i}-\bar{X}}{\sigma_{X}}$ is the standardized score of the metric sample $X_{i}$, $\bar{X}$ is the average of the metric samples $X_{i}$, and $\sigma_{X}$ is the sample standard deviation of the metric samples $X_{i}$. Similarly, $\frac{Y_{i}-Y}{\sigma_{Y}}$ is the standardized score of the metric sample $Y_{i}$, $\bar{Y}$ is the average of the metric sample $Y_{i}$, and $\sigma_{Y}$ is the sample standard deviation of the metric sample $Y_{i}$. The fluctuation range of the PPMCC of the two metrics samples is [−1,1]. γ=1 indicates that the metric samples $X_{i}$ and $Y_{i}$ have a good correlation, can be characterized by linear equations, and are positively correlated; γ=−1 indicates that the metric samples $X_{i}$ and $Y_{i}$ have a good correlation, can be characterized by linear equations, and are negatively correlated; and γ=0 indicates that the metrics do not have a linear relationship with each other.*

**Figure 7:**
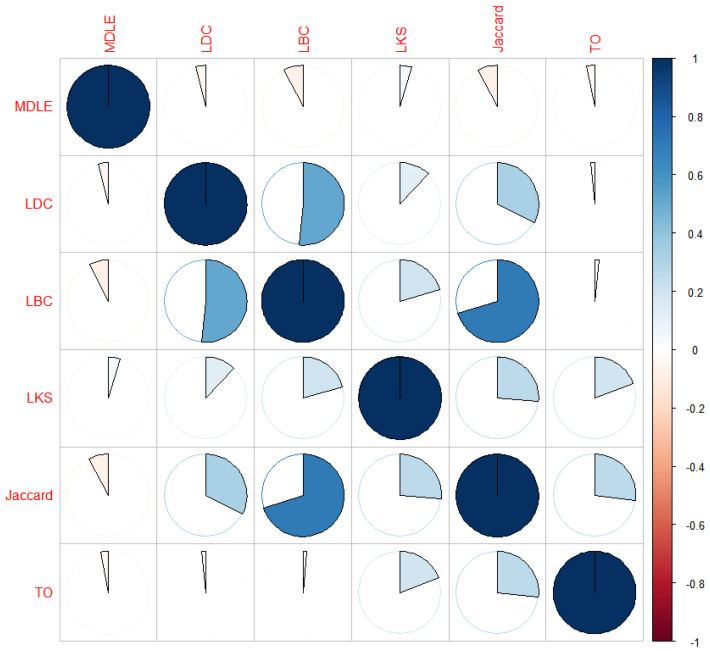
PPMCC heat map of link importance metrics.

In this section, we also evaluate the network topology based on the “Business” dataset, calculate the Pearson correlation coefficients between the MDLE and the five link importance metrics, and map the results into a heat map as shown in Figure 7.

In Figure 7, the heat map characterizes the strength of the correlation between the two metrics in terms of the percentage of fill of the circular pattern and the positive and negative correlation between the two metrics in terms of the color mapping of the legend on the right-hand side, thus providing a graphical presentation of the Pearson coefficients between the metrics. The diagonal line in the figure is the autocorrelation of each indicator, represented by a filled dark blue circular pattern (i.e., γ=1), and the lower left part of the figure is symmetric with the upper right part.

Based on the analysis of the heat map values and color distribution, it can be seen that, generally, the correlation between different assessment metrics presents the following characteristics: First, for the set of homogeneous metrics (link importance is either positively or negatively correlated with the metric values), the metrics are positively correlated with each other; this is because the critical links in the network often take on important functions in multiple dimensions. However, the correlation strength between them fluctuates due to the different assessment perspectives of the link importance metrics, e.g., the correlations between LDC, LBC, LKS, Jaccard, and TO are positive, but the magnitudes of the values of the Pearson parameter are different. It is worth noting that the assessment perspectives of various link importance metrics differ. However, the experimental network’s topology has an impact on the assessment results to some extent. For instance, while the LBC and Jaccard index assess links based on connectivity and endpoint variability, respectively, there are positive and strong correlations in the assessment results because the physical topology layer of the network presents a structure with solid centrality and leafy distribution at the edges. As a result, the LBC’s assessment results exhibit a strong correlation with the Jaccard index’s assessment results.

Meanwhile, the analysis shows that the correlation between the MDLE and the other five metrics is weakly negative. The reason why the correlation performance is different from the general indicators can be analyzed from the following two aspects.

First, from the perspective of the structural characteristics of the network model, the network security situational awareness-oriented multi-layer network model, the importance of the link not only depends on the topological location, but is also affected by the dependency relationship in the network. As a directional-dependent link fails, the dependent nodes associated with it will undergo secondary failure, which will lead to network damage on a larger scale. The existing link importance indicators only consider the single-layer network information, and cannot consider dependency as an assessment element, so it is difficult to find the set of links that have a significant impact on the three-tiered network topology.

The second is the idea of critical link discovery, where the MDLE aggregates the importance information in the three-layer network topology through the dependencies between network layers, can measure the importance of links from a domain-wide perspective, and can discover the set of links that play an important role in supporting the entire network topology. Some links are replaceable in the network and although they are located near the central area of the network, for instance, router communication links, in the event of a failure, the connectivity function can be replaced by a similar link with less impact on the network. On the contrary, failure of the links between the incoming routers and the important servers will make the servers go offline, which will have a great impact on the upper-layer service flow and a large amount of users’ access behaviors. These important links are often non-important in existing importance metrics. Meanwhile, the MDLE determines the importance of a network by evaluating its impact on all three tiers. As a result, it has a weak negative correlation with the current link importance metrics in terms of relevance.

### 5.4. Link-Removal-Based Network Destruction Simulation

In the link removal experiments, the node failure ratio (labeled θ) and link failure ratio (labeled ζ) of the three-layer network are utilized to gauge the level of influence that link removal has on the network’s structural integrity. The preferred node failure ratio is defined in the following manner.

**Definition** **17** (Node Failure Ratio).

(15) $$\theta =\frac{N_{\mathrm{PD}}^{\mathrm{Loss}}+N_{\mathrm{BA}}^{\mathrm{Loss}}+N_{\mathrm{UR}}^{\mathrm{Loss}}}{N_{\mathrm{PD}}+N_{\mathrm{BA}}+N_{\mathrm{UR}}}$$

*In Equation (15), $N_{\mathrm{PD}}$, $N_{\mathrm{BA}}$, and $N_{\mathrm{UR}}$ denote the number of failed nodes in the entire network topology in the physical device layer, the business application layer, and the user role layer, respectively. $N_{\mathrm{PD}}^{\mathrm{loss}}$, $N_{\mathrm{BA}}^{\mathrm{loss}}$, and $N_{\mathrm{UR}}^{\mathrm{loss}}$ denote the number of failed nodes in the physical device layer, the business application layer, and the user role layer, respectively, in the experimental process.*

Similarly, the link failure ratio is defined as follows.

**Definition** **18** (Link Failure Ratio).

(16) $$\zeta =\frac{L_{\mathrm{PD}}^{\mathrm{loss}\;}+L_{\mathrm{BA}}^{\mathrm{loss}\;}+L_{\mathrm{UR}}^{\mathrm{loss}\;}}{L_{\mathrm{PD}}+L_{\mathrm{BA}}+L_{\mathrm{UR}}}$$

*In Equation (16), $L_{\mathrm{PD}}$, $L_{\mathrm{BA}}$, and $L_{\mathrm{UR}}$ denote the number of failed links in the entire network topology in the physical device layer, the business application layer, and the user role layer, respectively. $L_{\mathrm{PD}}^{\mathrm{loss}}$, $L_{\mathrm{BA}}^{\mathrm{loss}}$, and $L_{\mathrm{UR}}^{\mathrm{loss}}$ denote the number of failed links in the physical device layer, the business application layer, and the user role layer, respectively, in the experimental process.*

Compared with the proportion of network node failure, the proportion of network link failure highlights the retention of links in the current subject network after the perturbation, which demonstrates the connectivity density of the network.

**Figure 8:**
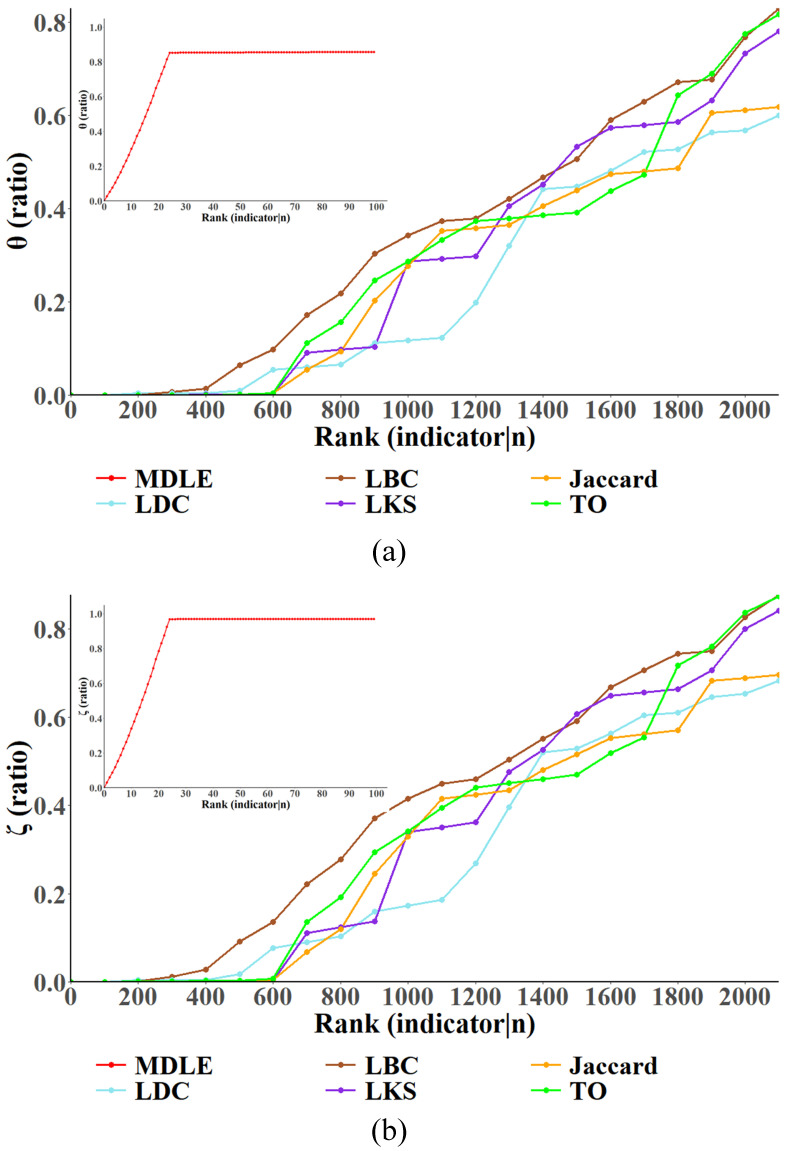
Relationship between link removal size and network node/link failure ratio (continuous series)—Business. This figure depicts the variation in node (subfigure (**a**))/link (subfigure (**b**)) failure ratio when links are successively deleted in the network "Business" ranked by link importance.

**Figure 9:**
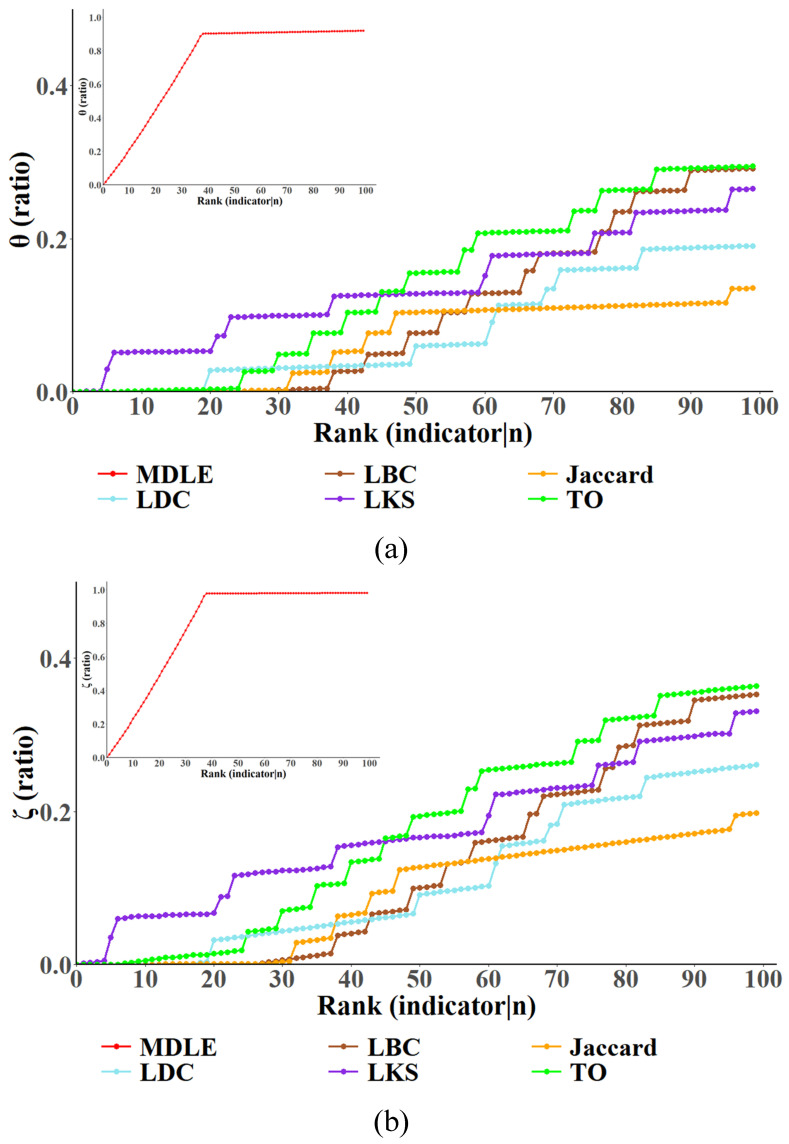
Relationship between link removal size and network node/link failure ratio (continuous series)—Campus. This figure depicts the variation in node (subfigure (**a**))/link (subfigure (**b**)) failure ratio when links are successively deleted in the network "Campus" ranked by link importance.

In this section, the continuous link removal strategy is used to conduct experiments, and the network node failure ratio and network link failure ratio of the “Business” and “Campus” networks are determined to observe the impact of link removal on the topology of the three-layer network model according to different link importance evaluation indexes. The results of the experiments are shown in Figure 8 and Figure 9.

The horizontal coordinate shows both the significance ranking and the size of the link that has been removed through the critical link discovery methodology; a higher ranking indicates that the link has a higher score of importance in the method, and also earlier the link is removed Campus in the experiment. The vertical coordinate characterizes the proportion of network node/link failures, which is identified by a separate subgraph, since the experimental results indicate that the MDLE has a significant advantage in the speed of convergence compared to the other link importance metrics.

To analyze the validity of the MDLE more comprehensively, this section of the experiment employs two comparison strategies for the “Business” and “Campus” networks: (a) In the “Business” network removal experiment, after the MDLE-based destruction strategy makes the network node/link failure ratio reach the convergence, the convergence position of the failure ratio is recorded, and then link removal is carried out based on the existing link importance index until the network failure ratio is close to the convergence position in the MDLE and the scale of link removal in the network at this time is counted. (b) In the “Campus” network removal experiment, a set of links with the same size is selected based on their importance metrics (including the MDLE), specifically the top 100 links per metric. These links are then removed and the experiment observes the percentage of network node/link failures.

In Figure 8, it is shown that during the link removal experiments, the convergence position of the MDLE in the percentage of network node failures and the percentage of link failures is around 85% and 97%, respectively. The experimental results show that when the two best metrics of network damage in the comparison methods, the link betweenness centrality (LBC) and the topology overlay index (TO), converge, the size of the removed links in the network has already exceeded 2000, while the MDLE discovery method reaches the convergence of the network node/link failure ratio before removing 30 links. This proves that the MDLE, which integrates multi-layer information, has a significant advantage in the discovery of critical links in multi-layer networks.

In Figure 9, with the increasing scale of link removal in the network, the proportion of node/link failure in the network increases positively, and the experimental results show that compared with the other five metrics (LDC, LBC, LKS, Jaccard, and TO), the MDLE can make the proportion of node failure in the network converge quickly. After removing the top 100 links, the proportion of node failure in the network node removal based on the five existing link importance assessment metrics is around 10–30%, while the effect of removal based on the MDLE reaches more than 90%. Similarly, assessing from the perspective of the network link failure ratio, after removing the top 100 links ranked by each metric, for the link removal strategy based on the existing link importance assessment metrics, the network link failure ratio is about 2–40%, while the effect of removal based on the MDLE is close to 100%, which means that connectivity has been paralyzed in the three-layer network. The experimental results show that in the multi-layer network model oriented to network security situational awareness, compared with the existing link importance metrics, the link importance rankings in the MDLE are more reasonable, and the MDLE can be more effective in discovering the critical link sets in the three-layer network.

## 6. Conclusions

By analyzing the architecture of a multi-layer network model and the distribution of links, this paper proposes a link importance assessment metric: the multi-layer-dependent link entropy (MDLE). This metric synthesizes the link-local betweenness mapping entropy and the dependent mechanism for critical link discovery. Since the existing link discovery methods cannot better solve the problems of a poor link information completeness and a huge link scale in networks, we construct a topology analysis domain centered on the target link, calculate the local betweenness centrality, and enhance the evaluation effect based on the mapping entropy theory. At the same time, decomposing the coupling relationship of links in the first-order central domain, combined with the link-dependent mechanism, enables the importance of links to be transmitted in the multi-layer network model, thus completing the convergence of importance metrics in each network layer and discovering the set of links that play a key role in the network. In the experimental portion, we employed different link importance metrics, including the Jaccard index, as a basis for comparison. The outcomes of the ranking monotonicity tests demonstrate that the MDLE is more precise in evaluating the granularity of link importance metrics within the network. After conducting a similarity analysis using PPMCC-based metrics, it was discovered that the link assessment results produced by the MDLE with the gain-dependency mechanism differed from the results obtained from single-layer information metrics. The MDLE results were found to be better suited for multi-layer structural characteristics. Finally, the results of the link removal experiments show that, compared to the five link importance metrics for which control experiments were conducted, the ranking results of the MDLE are more reasonable, with significant advantages in both the node failure ratio and link failure ratio results. The larger the network size, the more superior the assessment becomes.

Currently, our team has made some progress in building multi-layer network models for network security situational awareness. We have completed several tasks such as model building, critical node identification, and critical link discovery. These works have been covered in our previous works and this paper. As a result, we have established a basic research framework. The next step is to build upon this foundation and continue to develop our understanding of network security situational awareness. However, regarding the actual demands of network security situational awareness systems, the above work mainly improves the effectiveness of situational assessments from the network topology perspective, while in an actual network environment, dynamic changes in service traffic and the importance of user role information will further expand the complexity of situational assessments. Therefore, in future research, based on the multi-source information support and complex architecture support provided by multi-layer networks, the introduction of knowledge graph [36] technology to portray threat behaviors or the combination of large language models [37] to assist in processing massive situational data will help to further enhance the perception and assessment of the overall security position of a network. 

## Figures and Tables

**Table 1:** Link connection type catalog.

**Connection** **Type**	**Point 1**	**Point 2**	**Pos**	**Description**
**Connected** **Link**	route	route	intra	Communication relationship between routers
	business	business	intra	Business linkages or calling relationships between business systems
	business	access	intra	Access relationships between access nodes and business systems
	access	user	inter	Associations for user accounts logging in from access nodes
	user	user	intra	Social relationships or business connections between user roles
**Connection** **Type**	**Support** **Point**	**Dependent** **Point**	**Pos**	**Description**
**Dependent** **Link**	route	server	intra	Server is dependent on the communication support provided by the router
	route	terminal	intra	Terminal is dependent on the communication support provided by the router
	server	business	inter	Business system is dependent on the environment provided by server
	terminal	access	inter	Access node is entity mappings of a terminal in the business access relationship
	business	user	inter	User roles are groups of accounts that belong to the business system

## Data Availability

The [Network Topology] data used to support the findings of this study have been deposited in the [muti-layer-network] repository ([https://github.com/multilayer-go/muti-layer-network], accessed on 5 December 2023).
